# Engineered NLS-PBase system boosts stability and productivity in recombinant cell lines

**DOI:** 10.1186/s40643-026-01056-x

**Published:** 2026-04-28

**Authors:** Yanan Zhou, Yuan Tian, Qingyuan Ran, Qian Ye, Wen-Song Tan

**Affiliations:** 1 State Key Laboratory of Bioreactor Engineering, East China University of Science and Technology, 130 Meilong Road, Shanghai, 200237 People’s Republic of China; 2 Shanghai BioEngine Sci-Tech CO., LTD, 3F&4F Building 3, 396 Lvzhou Ring Road, Minhang District, Shanghai, 201203 People’s Republic of China

**Keywords:** Stability, Cell line construction, Transposon, PiggyBac system, Nuclear localization signal, Production

## Abstract

Chinese hamster ovary (CHO) cells constitute the industry-standard platform for the production of complex therapeutic proteins, yet genomic heterogeneity arising from random integration leads to clonal variability and unstable expression, necessitating a robust cell line development process to efficiently isolate stable, high-expressing clones. By employing an optimized integration strategy, recombinant cell line performance can be enhanced through improved genomic stability and increased productivity. In this study, GFP reporter analysis demonstrated that the PiggyBac system significantly boosts the yield of both stable and high-expression clones. Subsequently, transposase modified with a nuclear localization signal (NLS) exhibited superior stability in recombinant cell lines and polyclonal pools. The nucleoplasmin NLS resulted in transgene integration into genomic loci that promote enhanced and more stable expression, thereby improving clonal distribution. Furthermore, this strategy also increased recombinant mAb expression by 97% in pools while enhancing average and specific productivity in derived cell lines. Additionally, recombinant suspension cells generated with the optimized system exhibited comparable performance, with over 50% of minipools showing robust growth. Overall, these findings highlight the promising utility of the NLS-optimized PiggyBac system for improving transgene stability and expression while streamlining the cell line screening process.

**Graphical abstract:**

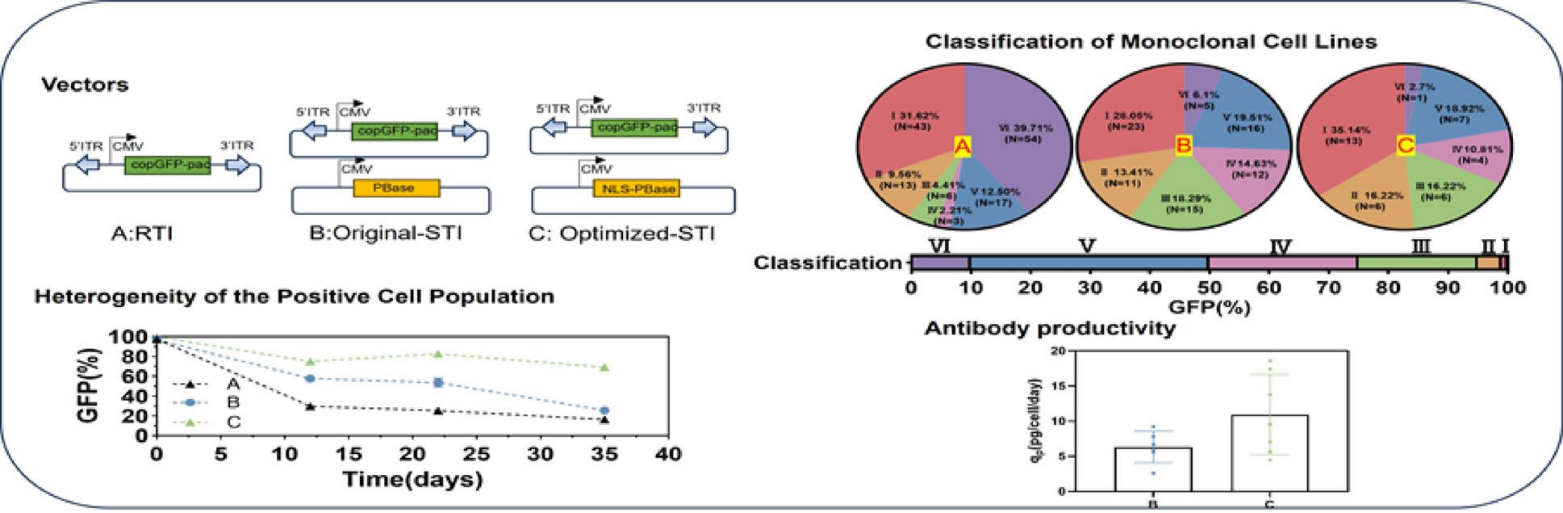

**Supplementary Information:**

The online version contains supplementary material available at 10.1186/s40643-026-01056-x.

## Introduction

Mammalian cells dominate therapeutic protein production owing to their accurate post-translational modification capabilities (Wurm 2004). As the industry workhorse, CHO cells account for 89% of biologics produced using mammalian systems (Walsh and Walsh 2022) and contribute to nearly half of the top-selling therapeutics in 2024 (Verdin 2025). The development establishment of recombinant CHO cell lines relies on genomic integration of both the gene of interest and a selectable marker to enable the cell enrichment followed by screening for high-producing clones. Central to this process are the integration method and screening efficiency, which directly determine the yield of stable, high-expressing clones. Optimization of selection stringency is widely applied to eliminate low-producing clones (Balasubramanian et al. 2016), yet upstream advances in genomic integration remain the most decisive factor in overall productivity.

Current transgene integration strategies can be broadly categorized by integration mechanism: random transgene integration (RTI), semi-targeted transgene integration (STI), and site-specific transgene integration (SSI). RTI relies on a non-homologous end joining–mediated process, generating diverse genomic outcomes that fundamentally restrict long-term clonal stability (Gu et al. 1992; Kromenaker and Srienc 1994; Zeyda et al. 1999; Pilbrough et al. 2009) and necessitate extensive screening to recover a small fraction of desirable clones. SSI employs engineered nucleases (e.g., CRISPR/Cas, TALENs, ZFNs) (Yunxiao Ren 2019) to direct precise insertion into pre-validated genomic safe harbors such as *C12orf35* (Ritter et al. 2016; Zhao et al. 2018), *Hprt* (Koyama et al. 2006; Wang et al. 2017; Kawabe et al. 2018), and *FER1L4* (Inniss et al. 2017), significantly enhancing clonal stability (Li et al. 2018; Ng et al. 2021). However, the limited number of nuclease-accessible loci generally restricts SSI to one or two transgene copies per genome, imposing an intrinsic expression ceiling.

STI approaches like transposon-based systems or viral transduction represent an attractive middle ground, enabling targeted insertion into transcriptionally active regions while permitting multi-copy integration. Among them, DNA transposons such as PiggyBac (PB), Sleeping Beauty, and Tol2 have gained considerable interest due to their high cargo capacity and reproducible integration profiles (Martin Munoz-Lopez and Jose L. Garcia-Perez 2010). PB, originally isolated from the cabbage looper moth (*Trichoplusia ni*), contains 13-bp inverted terminal repeats (ITRs) flanking a 2,472-bp transposon and encodes a 594-amino-acid transposase (PBase) (Cary et al. 1989; Fraser et al. 1995). Codon optimization and targeted amino acid mutations of the PBase significantly enhance transposition efficiency (Cadiñanos and Bradley 2007; Yusa et al. 2011). Importantly, the PBase exhibits precise targeting of TTAA sites in genic regions and regulatory hotspots, including transcription start sites, CpG islands, DNase I hypersensitive sites, and regions adjacent to long terminal repeats (Wilson et al. 2007; Galvan et al. 2009), which underlies the superior productivity and stability of PB-derived CHO cell lines (Mattia Matasci et al. 2011; Balasubramanian et al. 2015; Huhn et al. 2023). Despite these advantages, the inherently locus-agnostic functionality of PBase and its inefficient active nuclear import remain critical barriers to achieving predictable and tunable transposition outcomes. The NLS, which mediates protein import into the nucleus via the importin α/β1 pathway, has emerged as a versatile tool for nucleic acid and protein delivery engineering (Smith et al. 1997; Efthymiadis et al. 1997; Jans et al. 2000). Fusion of NLS motifs to genome-editing tools such as CRISPR-Cas9 markedly enhances nuclear localization and editing efficiency (Park et al. 2022). Nevertheless, within cell line development, the relationship between NLS-mediated nuclear transport and transgene stability and expression has not been systematically dissected. Among the identified NLS (Hicks 1995; LaCasse and Lefebvre 1995), the most extensively used and well-characterized types are the monopartite and bipartite NLS. The SV40 large T-antigen NLS (PPKKKRKV) exemplifies the monopartite type, whereas the bipartite NLS comprises two clusters of basic residues separated by a linker peptide, as seen in nucleoplasmin (KRPAATKKAGQAKKKK) from *Xenopus laevis* (Dingwall and Laskey 1991), c-Myc (PAAKRVKLD), and Mc-myc (PAAKKKKLD).

In this study, we employed a fluorescent reporter-based system combined with clonal distribution analysis to systematically evaluate cell line stability and expression. Our analysis revealed significant limitations of the RTI method, most notably the unstable of clones. To overcome the lack of stable clones, this study aimed to enhance cell line development process based on PB system. The fusion of NLS to PBase was performed to increase cell line stability and expression. Furthermore, the superior NLSs were applied to produce monoclonal antibody (mAb) to assess the productivity of both the recombinant cell pool and the derived clonal cell lines. Additionally, correlations among gene copy number, transcript level, GFP mean fluorescence intensity (GFP-MFI), and productivity were established across multiple clones, revealing key predictive indicators of clonal performance. Overall, incorporating NLSs into the PB system enhances the cell line development workflow by increasing stable and high-expressing clones while reducing the labor required for screening.

## Materials and methods

### Cell line and cell culture

The adherent CHO-K1 cell line (ATCC, Virginia, USA; No. CCL-61) used in this work was cultured in Dulbecco’s modified Eagle’s medium (DMEM; Bioengine, Shanghai, China) supplemented with 10% (v/v) of fetal bovine serum (FBS; ExCell Bio, Suzhou, China), 0.069 g/L L-proline and 3.7 g/L NaHCO₃ in static T flasks (TPP, Trasadingen, Switzerland) at 37 ℃ with 5% CO_2_ atmosphere.

Suspension-adapted CHO-K1 cells were routinely cultured in Eden B601S medium (Bioengine) supplement with 6-8mM L-glutamine in TubeSpins (TPP) at 37 ℃, 5% CO_2_ atmosphere with a shaking frequency of 220 rpm. The suspension cells were routinely sub-cultured twice per week at a density of 3 × 10^5^ cells/mL. The cell density and viability were assessed by a cell counter (Countstar, Shanghai, China) based on the trypan blue staining method.

### Plasmids

**Fig. 1:**
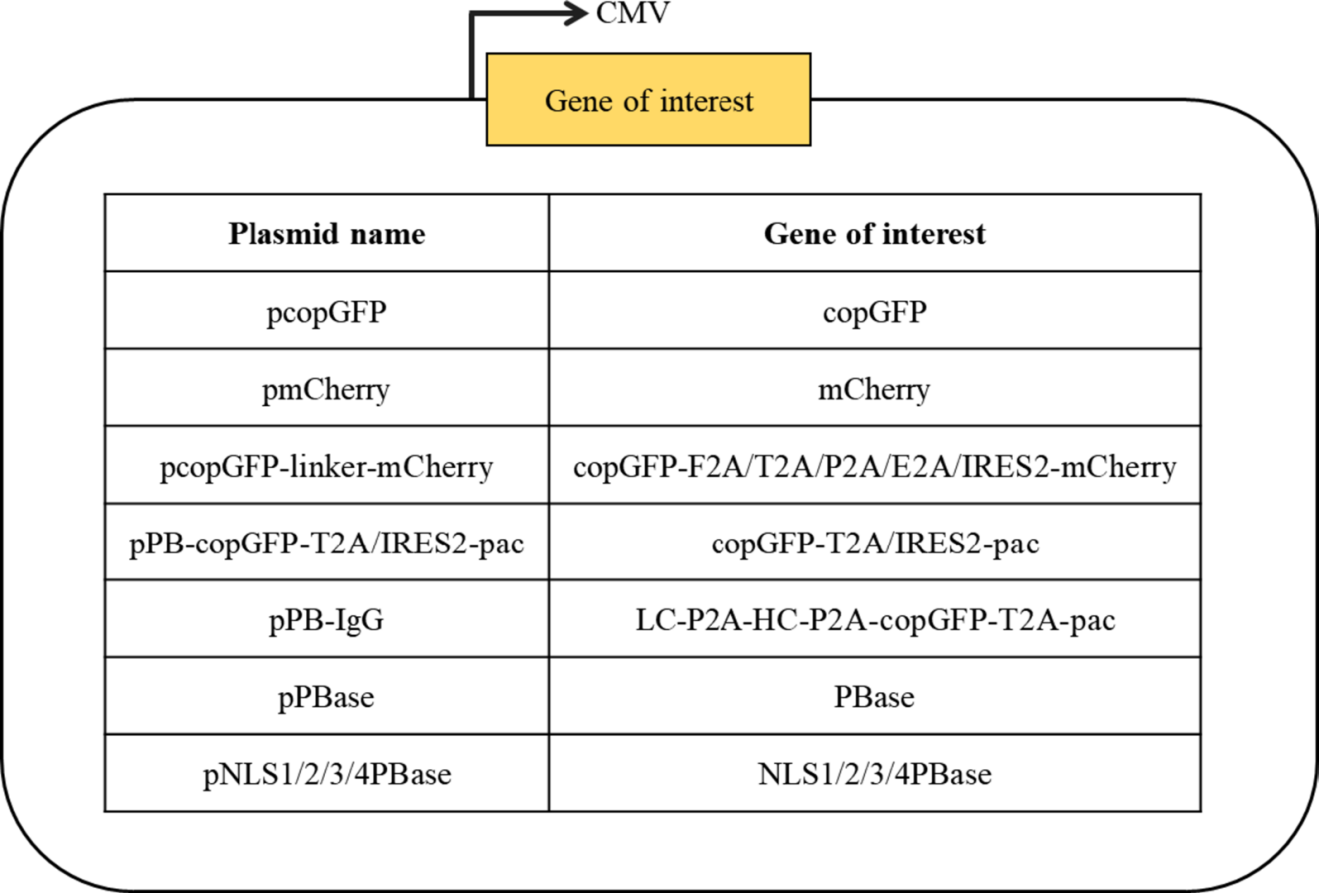
The plasmids were constructed into pcDNA3.1(+) backbone to express the copGFP, mCherry, LC, HC or PBase genes, each under the control of a CMV promoter. Abbreviations: CMV (mouse cytomegalovirus major immediate early promoter/enhancer); copGFP (Codon-Optimized Green Fluorescent Protein gene); mCherry (monomeric Cherry fluorescent protein gene); F2A/T2A/P2A/E2A (2 A peptides); IRES2 (internal ribosomal entry site 2); pac (puromycin N-acetyl-transferase gene); PBase (PiggyBac transposase); NLS (nuclear localization signal)

The pcopGFP or pmCherry vectors were constructed by cloning the Codon-Optimized Green Fluorescent Protein (copGFP) gene or monomeric Cherry fluorescent protein (mCherry) gene, respectively, into the pcDNA3.1(+) backbone under the control of the cytomegalovirus immediate-early gene promoter/enhancer (CMV). All of the following plasmids were constructed into the pcDNA3.1(+) backbone, unless otherwise specified. The pcopGFP-linker-mCherry vector was constructed by cloning a bicistronic expression cassette, which comprises the copGFP and mCherry genes, both placed downstream of a single CMV promoter. Their expression is facilitated by one of several intercistronic linkers: a self-cleaving 2 A peptide (F2A, T2A, P2A, or E2A), or the internal ribosome entry site 2 (IRES2) element. The vector pcopGFP+mCherry, containing both copGFP and mCherry genes each under the control of a separate CMV promoter, was provided by our laboratory. The donor vector pPB-copGFP-T2A/IRES2-pac was constructed featuring a PB transposon cassette flanked by ITRs. Within this cassette, the copGFP and puromycin resistance (pac) genes are expressed from a single transcript and separated by either a T2A self-cleaving peptide or an IRES2 element. To engineer the pPB-IgG construct, the heavy-chain (HC) and light-chain (LC) antibody cDNAs were inserted into the pPB-copGFP-T2A-pac, thus enabling the expression of HC, LC, GFP, and *pac* genes from a single polycistronic mRNA. The helper plasmid pPBase was constructed by cloning the PBase gene, amplified from PB200PA-1 (System Biosciences, CA, USA), into the pcDNA3.1(+) backbone. Previous research has demonstrated that PBase activity remains unaffected by protein fusion at either the N- or C-terminus (Owens et al. 2012). Four optimized helper plasmids were constructed by individually cloning sequences encoding four distinct NLSs-SV40 (NLS1), Nucleoplasmin (NLS2), c-Myc (NLS3), and Mc-Myc (NLS4)- into the pPBase vector, generating the corresponding constructs pNLS1PBase to pNLS4PBase. Schematic representations of the plasmid constructs are shown in Fig. 1.

### DNA transfection

For adherent cells, cells were seeded in 24-well plates with the cell density of 2 × 10^5^ cells/mL before 24 h of transfection. Transfection was performed using the Lipofectamine™ 8000 transfection reagent (Beyotime, Shanghai, China) according to the manufacturer’s instruction.

For suspension cells, cells were passaged to a density of 1–1.5 × 10^6^ cells/mL on the day before transfection. Electroporation was performed when cell density reached 2–4 × 10^6^ cells/mL with viability ≥ 95%, after which the cells were transferred into a 6-well plate containing 2 mL of basal medium.

### Flow cytometry

GFP and RFP fluorescence were measured with flow cytometer (Beckman Coulter, California, USA). Cells were collected 48 h post-transfection and resuspend in phosphate-buffered saline (PBS) for twice. Each sample was measured in duplicate with a minimum of 10,000 recorded events.

### Generation of recombinant cell pools and cell lines

For adherent cells, cells were co-transfected as described above, using either the donor plasmid alone (0.45 µg) or a donor-to-helper plasmid mixture at a 9:1 ratio (w/w). Antibiotic selection was initiated 48 h post-transfection using puromycin (Beyotime, Shanghai, China) at concentrations of either 5 µg/mL or 10 µg/mL and maintained until stable proliferation of the resistant cell population was achieved. Single-cell cloning was performed by standard serial dilution, seeding cells at a density of 0.5 cells per well in 96-well plates. After two weeks, clones were expanded into 48-well plates and analyzed by flow cytometry once confluence reached 50% or higher.

For suspension cells, cells were seeded at a density of 2,000 cells/well into 96-well plates and cultured statically under 10 µg/mL puromycin selection after 48 h post-electroporation. When confluence reached 30% or higher, cells were expanded into 48-well plates and subjected to flow cytometry analysis.

The diversity of cell pools was assessed by tracking the GFP-positive rate during one-month culture without antibiotic pressure. A higher GFP-positive rate served as an indicator of greater average cell line stability, reflecting sustained genetic and expression homogeneity over long-term culture. Cellular stability was evaluated by GFP-positive rate, with higher percentages correlating with enhanced stability in classified cell lines.

#### Quantification of mAb expression level

Mab concentration in adherent cell culture supernatants was directly determined by sandwich enzyme-linked immunosorbent assay (ELISA). Goat anti-Human IgG (H + L) (Thermo Fisher Scientific, Waltham, USA) was used for coating the ELISA-Plates and with HRP-conjugated Goat anti-Human IgG (H + L) (Thermo Fisher Scientific). Absorption was measured at 450 nm with a reference wavelength of 630 nm on a microplate reader (Thermo Fisher Scientific). Antibody content in the supernatants of suspension cells was quantified via affinity high-performance liquid chromatography (1260 HPLC system; Agilent Technologies, Santa Clara, USA) coupled with a BioCore Protein A column (NanoChrom, Beijing, China).

### Quantification of transgene copy number and mRNA expression

The total DNA and RNA were extracted from cells using the Genomic DNA Kit (Tiangen, Beijing, China) and TRNzol Universal RNA extraction kit (Tiangen) according to the manufacturer’s protocols. The first-strand cDNA was reverse-transcribed using FastKing cDNA First Strand Synthesis System (Tiangen). The relative copy number and mRNA level of transgene were estimated by real-time quantitative PCR (RT-qPCR) using the 2×Q1 SYBR qPCR Master Mix (TOLOBIO, Shanghai, China) on a CFX96 Touch™ system (Bio-Rad, California, USA). Oligonucleotide primers for RT-qPCR are listed in Table 1.

**Table 1:** Oligonucleotide primers used in this study for RT-qPCR

Genes	Forward primer (5′-3′)	Reverse primer (3′-5′)
IgG-LC	TTCGCAGGCGTAGACTTTGT	TGGATAACGCCCTCCAATCG
IgG-HC	TGAAGGGCCGATTCACCATC	TCGGACCAGTCAAAAGGTCG
GFP	GCGGATGATCTTGTCGGTGA	CCGCATCGAGAAGTACGAGG
*β-actin*	GGCTTTTGGAGTTTGACAATGC	CATGAGGTAGTCTGTCAGGTCCC

### Reverse inference of cell-pool heterogeneity from clonal cell lines

The diversity of cell pools is determined by both the intrinsic stability of individual clones and their specific growth rates (µ). Based on their distinct growth rates, cell lines in categories I-VI were assigned corresponding µ, respectively (Table. S1). Hence, we employed the following approach to estimate the proportion of positive cells on a given day (The symbol µ denotes the specific growth rate, whether alone or subscripted).

$$ \begin{gathered} D_{{n,i}} = \frac{{D_{{n - 1,i}} \cdot e^{{\mu i}} }}{{\sum\nolimits_{{i = 1}}^{6} {D_{{n - 1,i}} } \cdot e^{{\mu i}} }} \hfill \\ GFP\left( \% \right)D_{n} = \sum\nolimits_{{i = 1}}^{6} {D_{{n,i \cdot Mean_{i} }} } \hfill \\ \end{gathered} $$

Where $\:{D}_{n,i}$ denotes the proportion of clone class *i* on D_n_; $\:{\mu\:}_{i}$ represents the specific growth rate of clone class *i*; $\:{e}^{{\mu\:}_{i}}$ represents its growth multiple per generation; and $\:{Mean}_{i}$ is the mean GFP positive rate for clone class *i*.

### Statistical analysis

Data analyses were performed using GraphPad Prism version10 (GraphPad Software, USA). Statistical analysis was performed using a two-tailed Student’s t-test (for two groups), and one-way or two-way ANOVA (for multiple groups), followed by Tukey’s post-hoc test for multiple comparisons. The error bars represent the standard deviations of three independent biological replicates. Significance levels are denoted as ^*^p < 0.05, ^**^p < 0.01, and ^***^p < 0.001. When multiple comparisons are performed, the additional symbols ^^^ and ^#^ are employed as markers for statistical significance, with the same significance levels applying.

## Results

### Linker selection for polycistronic systems

**Fig. 2:**
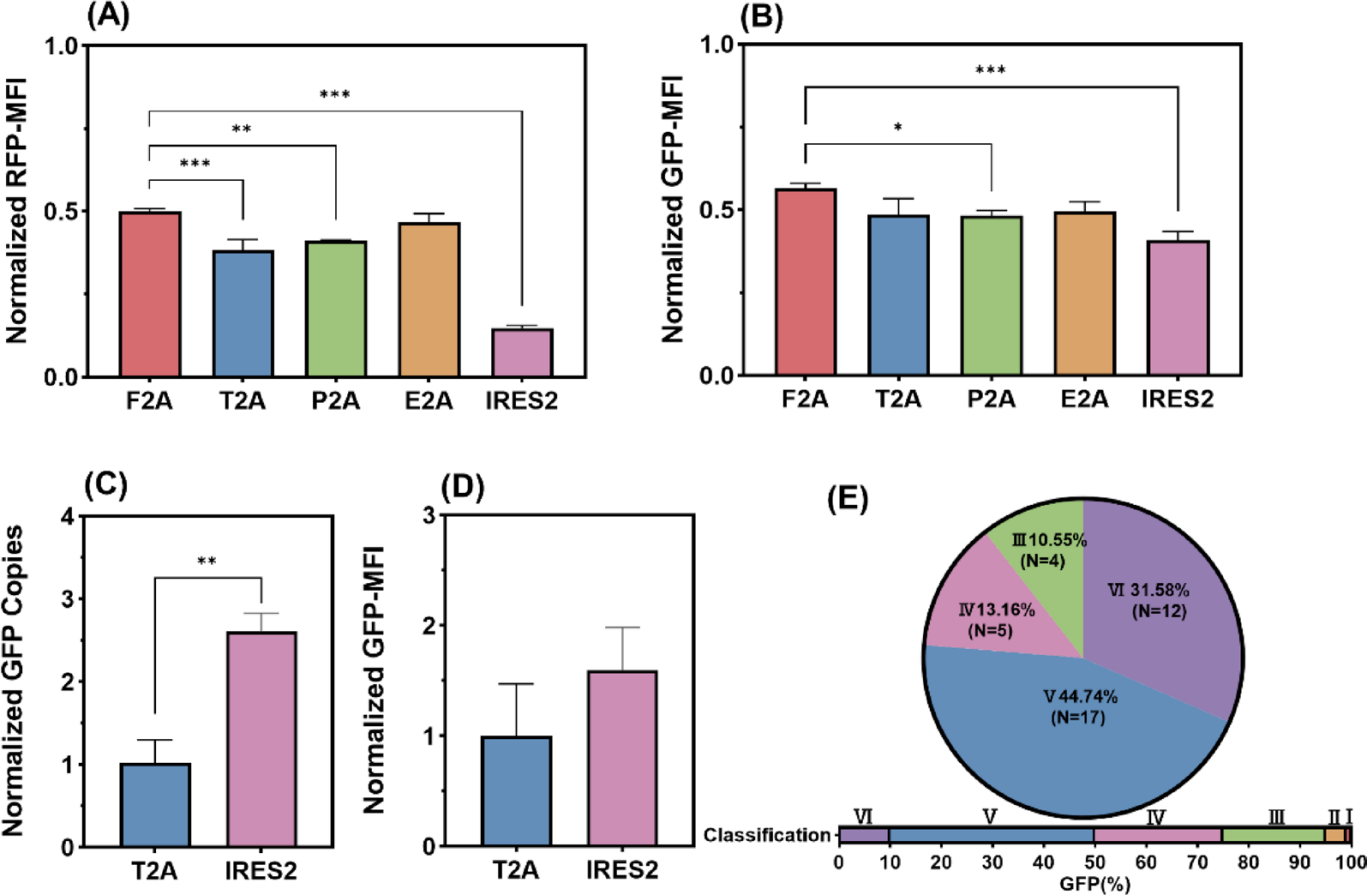
Linker selection for the establishment of polycistronic systems. Expression of RFP (**A**) and GFP (**B**) from pcopGFP-linker-mCherry construct after 48 h transient transfection, relative to the pcopGFP+mCherry control. Assessment of GFP copies (**C**) and GFP-MFI (**D**) in recombinant cell pools selected with 5 µg/mL puromycin, normalized to the T2A pool. **E** Categories of monoclonal cell populations. *P*-values were estimated by one-way ANOVA (A-T2A, P2A, E2A, and IRES2 vs. F2A: *p* < 0.001, *p* = 0.001, *p* = 0.28, *p* < 0.001; B-T2A, P2A, E2A, and IRES2 vs. F2A: *p* = 0.07, *p* = 0.03, *p* = 0.06, *p* < 0.001) and two-tailed Student’s t-test (C-T2A vs. IRES2: *p* = 0.002; D-T2A vs. IRES2: *p* = 0.17)

In polycistronic systems, self-cleaving 2A peptides (F2A, T2A, P2A, or E2A) and IRES2 function as linker sequences to coordinate the co-expression of multiple genes. We therefore first used a transient expression system to assess how different linkers affect the expression of the upstream (copGFP) and downstream (mCherry) genes. This was done using pcopGFP-linker-mCherry constructs, with the pcopGFP+mCherry vector serving as the control. Throughout this study, GFP and RFP refer to the Codon-Optimized Green Fluorescent Protein (originally copGFP) and the monomeric Cherry fluorescent protein (originally mCherry), respectively. Consequently, we selected a linker that showed minimal impact on GFP expression but substantial attenuation of RFP expression for linkage to the subsequent screening gene. The results showed that linking copGFP and mCherry genes led to differential attenuation of expression. Compared to control, GFP mean fluorescence intensity (GFP-MFI) decreased to 40.77%-56.48% of the control level, while RFP mean fluorescence intensity (RFP-MFI) was reduced to 14.86%-50.09% (Fig. 2A–B). Among the tested linkers, IRES2 produced the weakest fluorescence intensity for both GFP and RFP. By contrast, the T2A linker achieved moderate GFP expression while substantially reducing RFP output. Overall, bicistronic constructs connecting copGFP and the *pac* gene via T2A or IRES2 (pPB-copGFP-T2A/IRES2-pac) were transfected into CHO cells and selected with 5 µg/mL puromycin. While comparative genomic analysis showed that the IRES2 linker yielded a 1.6-fold higher copGFP gene copy number than T2A, this did not translate to a significant difference in GFP-MFI (Fig. 2C–D). During one month of culture in the absence of selection, both systems demonstrated comparable diversity and GFP-MFI in polyclonal populations (Fig. S1). Based on IRES2’s larger size (~ 600 bp vs. T2A’s ~ 60 bp) and the inverse correlation between plasmid size and transfection efficiency, T2A was selected for linking the resistance gene in stable cell line development.

Monoclonal cells sorted and expanded from the T2A-linked recombinant pool were classified into six categories based on their GFP-positive rates (%): Class I (99–100%), II (95–99%), III (75–95%), IV (50–75%), V (10–50%), and VI (0–10%). Analysis of the monoclonal cells revealed the absence of Class I and II clones, while the proportions of Class III, IV, V, and VI clones accounted for 10.55%, 31.58%, 44.74%, and 13.16%, respectively (Fig. 2E). The result indicates that conventional random integration, even when employing the optimized T2A linker, yield a low proportion of stable clones in the recombinant cell pool. The GFP-positive cell percentage dropped to merely 20–40% after 24 days culture (Fig. S1), which would significantly compromise cell line screening efficiency.

### The superiority of the PB system during stable cell lines construction

**Fig. 3:**
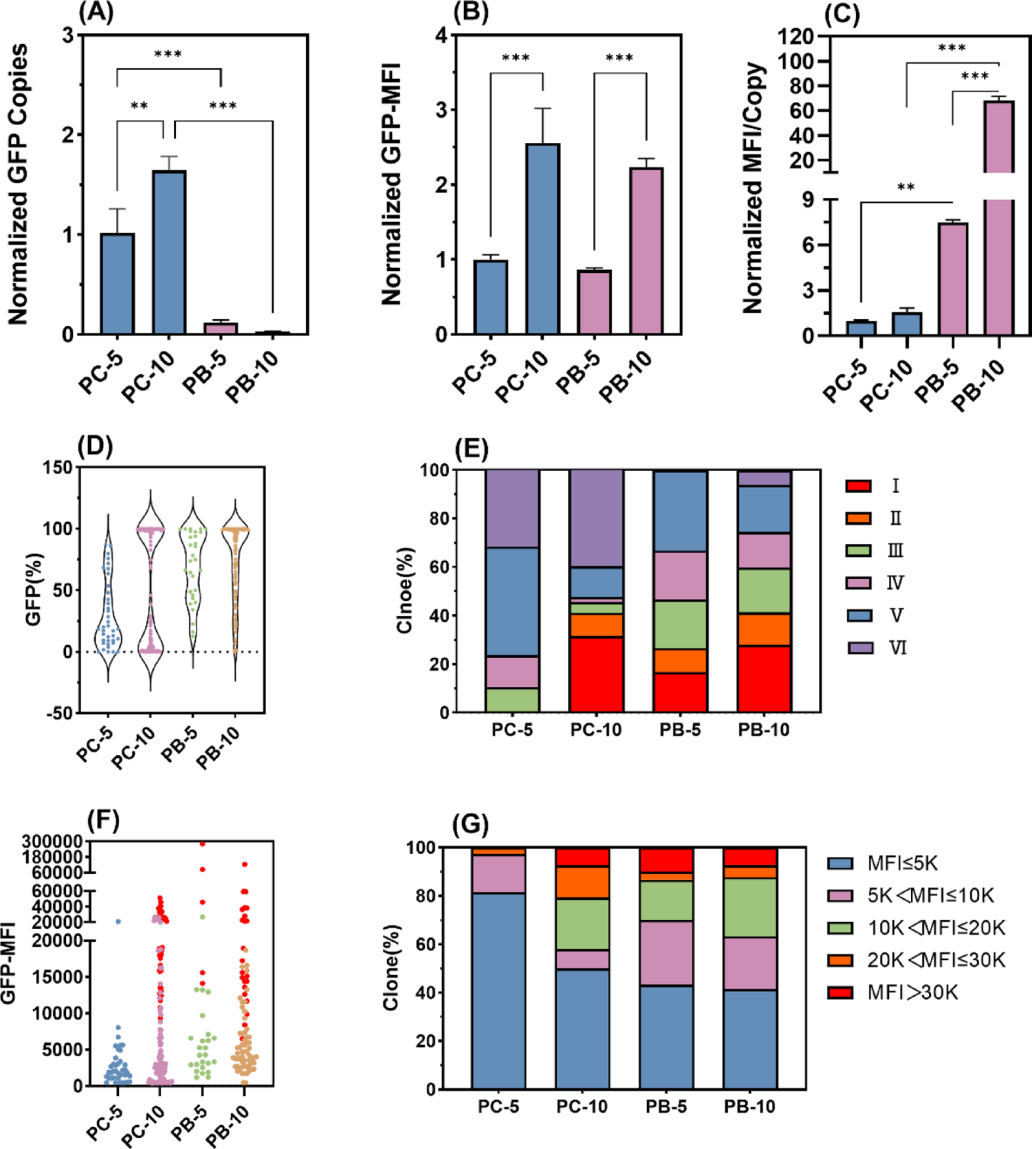
The influence of screening intensity and expression vectors on clonal stability and expression. The stable transfected recombinant cell pools established with pcDNA3.1 or PB at 5 µg/mL and 10 µg/mL puromycin are designated as PC-5, PC-10, PB-5 and PB-10, gene integration and expression were normalized to PC-5. **A** GFP gene copies, **B** GFP-MFI, **C** GFP-MFI/Copy, **D** The GFP positive cell ratio of the cell lines, **E** Proportion of clones belong to classes, **F** The MFI of positive cells, with red dots representing cell lines with GFP (%) ≥ 99%, **G** Proportion of clones with different expression levels in the recombinant cell pools. *P*-values were estimated by one-way ANOVA (A-PC-5 vs. PC-10: *p* = 0.006, PC-5 vs. PB-5: *p* < 0.001, PB-5 vs. PB-10: *p* = 0.93, PC-10 vs. PB-10: *p* < 0.001; B-PC-5 vs. PC-10: *p* < 0.001, PC-5 vs. PB-5: *p* = 0.90, PB-5 vs. PB-10: *p* < 0.001, PC-10 vs. PB-10: *p* = 0.40; C-PC-5 vs. PC-10: *p* = 0.98, PC-5 vs. PB-5: *p* < 0.010, PB-5 vs. PB-10: *p* < 0.001, PC-10 vs. PB-10: *p* < 0.001)

To improve the acquisition efficiency of stable clones (e.g., class I/II clones), the PB expression vector system was utilized with enhanced selection pressure to increase recombinant cell stability. GFP expression patterns were assessed in both recombinant cell pools and monoclonal derivatives under two selection pressures (5 µg/mL and 10 µg/mL puromycin), comparing the pcDNA3.1 (RTI) and PB (STI) expression systems. The results showed that 10 µg/mL puromycin pressure with the pcDNA3.1 system correlated with increased transgene copy numbers and a consequent rise in GFP expression. At 5 µg/mL puromycin selection, when replacing pcDNA3.1 with the PB transposon system, the overall expression level did not increase, but the copy number decreased while the GFP expression level per single copy increased. This observation is consistent with the reported characteristic of PB transposons preferentially integrating into transcriptionally active regions of the genome (Wilson et al. 2007). The PB transposon system under higher selection pressure boosting expression levels while lowering copy numbers-a strategic advantage underpinned by an 8.1-fold rise in per-copy expression that facilitates the acquisition of low-copy, high-expression clonal cell lines (Fig. 3A–C). Additionally, the percentage of GFP-positive cells remained at approximately 99% during selection culture (Fig. S2).

Further clonal analysis revealed that using the PB transposon system instead of pcDNA3.1 under 5 µg/mL puromycin increased Class I clones from 0% to 16.67%, whereas the combined proportion of Class V-VI clones decreased from 76.32% to 33.33%. Increasing PB selection pressure produced 28.05% Class I clones and notably reduced Class V/VI clones to 25.61% (Fig. 3D–E). Clonal cell lines were stratified by GFP-MFI into five categories: high (> 30 K), moderate-high (20–30 K), moderate (10–20 K), moderate-low (5–10 K), and low-expression (≤ 5 K). At 5 µg/mL puromycin, the PB system increased high-expression (> 30 K) and moderate-expression (10–20 K) clones from 0% to 10% and 16.67%, respectively, while reducing low-expression (≤ 5 K) clones from 81.58% to 43.33%. Enhanced selection pressure further raised moderate-expression (10–20 K) clones from 16.67% to 24.39% and decreased moderate-low-expression (5–10 K) clones from 26.67% to 21.95%. Although the PB system at 10 µg/mL puromycin did not increase Class I or high-expression (> 30 K) clones compared to pcDNA3.1, it significantly reduced Class V and VI clones from 52.21% to 25.61% and low-expression (≤ 5 K) clones from 50.00% to 41.46% (Fig. 3F–G). Therefore, employing the PB system or elevated selection pressure concurrently enhanced the yield of high-quality clones (Class I/II; high/moderate-high expression) while reducing low-performance populations (Class V/VI; low expression).

### The enhancement of NLS for cell stability and expression

To enhance recombinant cell stability, we generated four PBase variants by fusing the following NLS to its N-terminus: SV40 large T-antigen (NLS1), nucleoplasmin (NLS2), c-Myc (NLS3), and Mx-myc (NLS4). A no-NLS PBase construct served as the control.

**Fig. 4:**
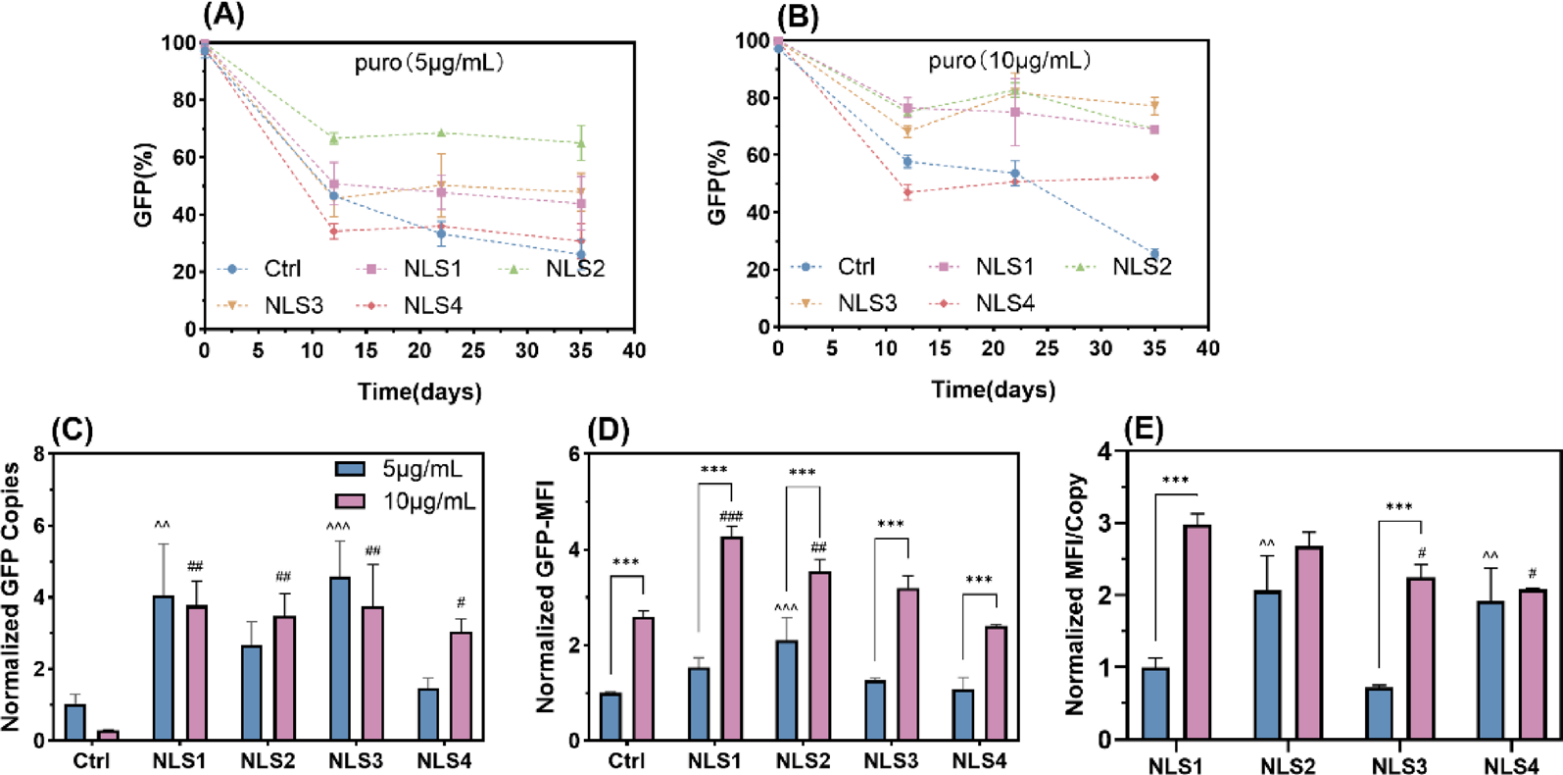
The effect of NLS on recombinant cell pools. Recombinant cell pools were obtained under selection with 5 µg/mL and 10 µg/mL puromycin, with the no-NLS group as the control. The positive cell analysis of recombinant cell pools enriched with 5 µg/mL puromycin (**A**) and 10 µg/mL puromycin (**B**). **C** GFP gene copies, **D** GFP-MFI, **E** GFP-MFI/Copy. *P*-values were estimated by two-way ANOVA(C-5 µg/mL vs. 10 µg/mL: Ctrl: *p* = 0.99, NLS1: *p* > 0.99, NLS2: *p* = 0.96, NLS3: *p* = 0.95, NLS4: *p* = 0.37; 5 µg/mL-NLS1, NLS2, NLS3, and NLS4 vs. Ctrl: *p* = 0.005, *p* = 0.31, *p* < 0.001, *p* > 0.99; 10 µg/mL-NLS1, NLS2, NLS3, and NLS4 vs. Ctrl: *p* = 0.004, *p* = 0.009, *p* = 0.004, *p* = 0.03. D-5 µg/mL vs. 10 µg/mL: Ctrl: *p* < 0.001, NLS1: *p* < 0.001, NLS2: *p* < 0.001, NLS3: *p* < 0.001, NLS4: *p* < 0.001; 5 µg/mL-NLS1, NLS2, NLS3, and NLS4 vs. Ctrl: *p* = 0.18, *p* < 0.001, *p* = 0.92, *p* > 0.99; 10 µg/mL- NLS1, NLS2, NLS3, and NLS4 vs. Ctrl: *p* < 0.001, *p* = 0.002, *p* = 0.08, *p* > 0.99. E-5 µg/mL vs. 10 µg/mL: NLS1: *p* < 0.001, NLS2: *p* = 0.14, NLS3: *p* < 0.001, NLS4: *p* > 0.99; 5 µg/mL-NLS2, NLS3, and NLS4 vs. NLS1: *p* = 0.003, *p* = 0.89, *p* = 0.009; 10 µg/mL-NLS2, NLS3, and NLS4 vs. NLS1: *p* = 0.84, *p* < 0.05, *p* = 0.01).^*^Represents significant analysis within the group; ^^^represents significant analysis between groups at 5 µg/mL; ^#^represents significant analysis between groups at 10 µg/mL

NLS2 conferred the greatest stability enhancement at 5 µg/mL puromycin, maintaining 65.00% positive cells after passaging, compared to 43.92% for NLS1 and 47.86% for NLS3 (Fig. 4A). Elevated selection pressure further enhanced recombinant cell stability. The post-passage positive rates reached 69.08% (NLS1), 69.08% (NLS2), 77.19% (NLS3), and 52.35% (NLS4) (Fig. 4B). At high selection pressure, NLS1 and NLS3 mediated a notable increase in integration copies, but this elevation did not translate to higher expression levels. In contrast, NLS2 did not show a significant increase in integration copy number, but its expression was markedly enhanced by 109.54% (Fig. 4C). Increased selection pressure boosted expression without altering integration copies, demonstrating improved functional efficacy on a per-copy basis (Fig. 4C–D). Per-copy GFP expression analysis revealed that NLS2 achieved the highest level under 5 µg/mL puromycin. When selection pressure increased, NLS1 and NLS2 showed the highest expression among all groups, respectively. The N-terminal fusion of NLS to PBase enhances both recombinant cell pool positive cells and transgene expression in the PB system. Among the tested sequences, NLS1 and NLS2 collectively demonstrate superior performance in stabilizing cell line and elevating per-copy expression.

**Fig. 5:**
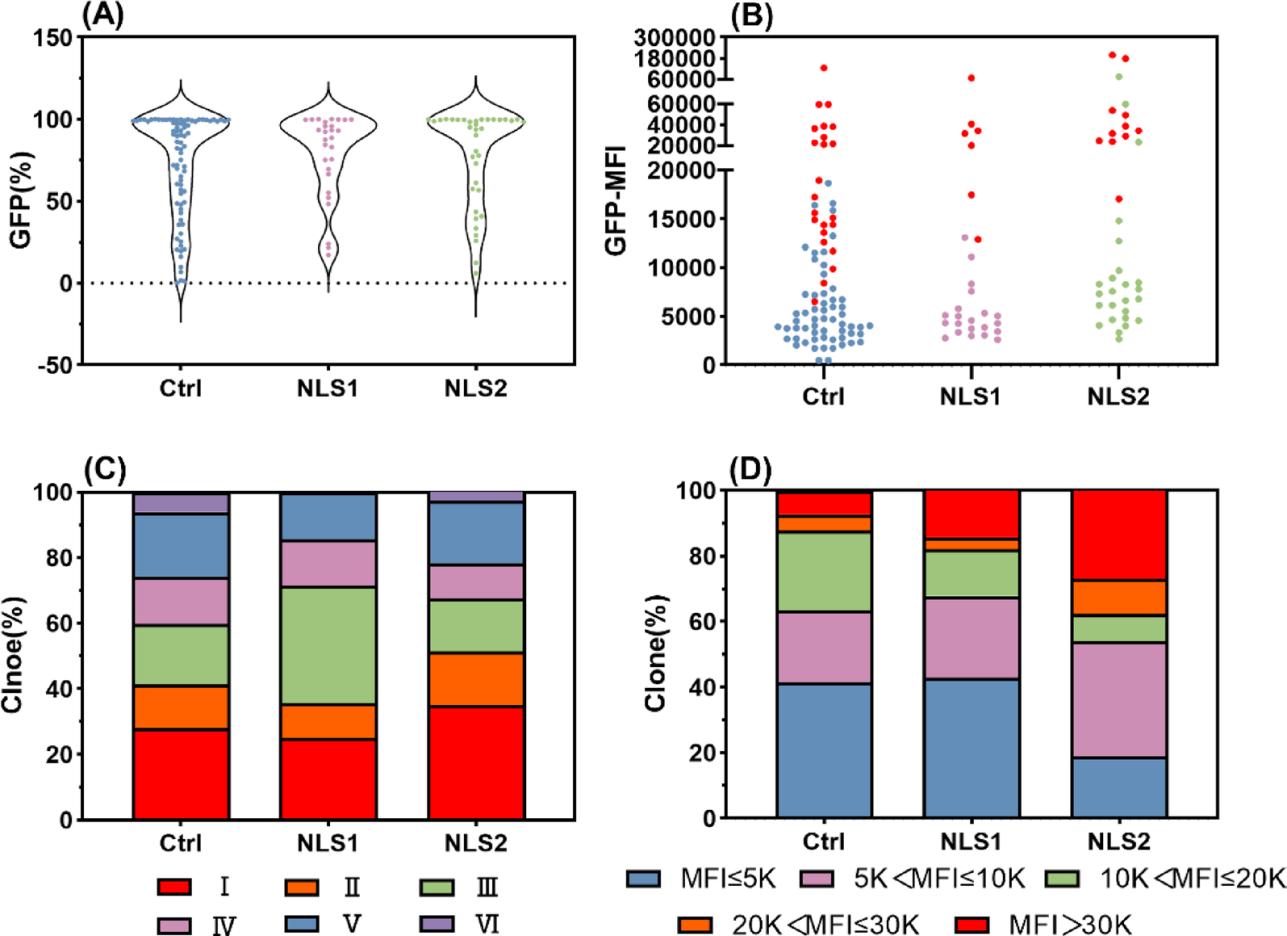
Stability and expression of clones isolated from NLS1 and NLS2 cell pools. Monoclonal cell lines were isolated from the recombinant cell pools obtained under 10 µg/mL puromycin selection, using no-NLS as the control group. **A** The positive cell ratio of the cell lines, **B** The MFI of positive cells, with red dots representing cell lines with GFP (%) ≥ 99%, **C** Proportion of clones belong to different classes, **D** Proportion of clones with different expression levels in the recombinant cell pools

Selection with 10 µg/mL puromycin confirmed the higher overall efficacy of the NLS fusions. Among all constructs, the NLS1 and NLS2 variants demonstrated the most robust cell pools and achieved the highest expression levels, both in terms of total output and on a per-copy basis. We subjected clonal isolates from these groups to comparative analysis against the no-NLS control (Fig. 5A–B). NLS1 application increased Class III clones from 18.29% to 25.00% and high-expression clones from 7.32% to 14.29%, while reducing Class V/VI clones from 25.61% to 14.29%. NLS2 further enhanced this trend, raising Class I clones from 28.05% to 35.14% and high-expression clones to 27.03%, while cutting low-expression clones from 41.46% to 18.92% (Fig. 5C–D). Furthermore, a strong correlation was established between the dynamic changes in the overall positive cell ratio of the recombinant cell pool and the derived cell lines. The observed clonal distribution showed strong statistical alignment with the predicted GFP (%) values, with all data points falling within the 95% confidence interval of the actual measurements except for PB-5 (Fig. S3).

Therefore, NLS1 and NLS2 enhance recombinant cell pool stability primarily by enriching Class I and depleting Class V/VI populations, while concurrently increasing high-expression variants. The GFP (%) of the cell pool has proven to be an effective predictor of clonal stability, providing a practical screening criterion that allows for more efficient stable clone isolation and a reduced experimental workload.

### Enhancement of mAb production by the optimized system in adherent CHO-K1

**Fig. 6:**
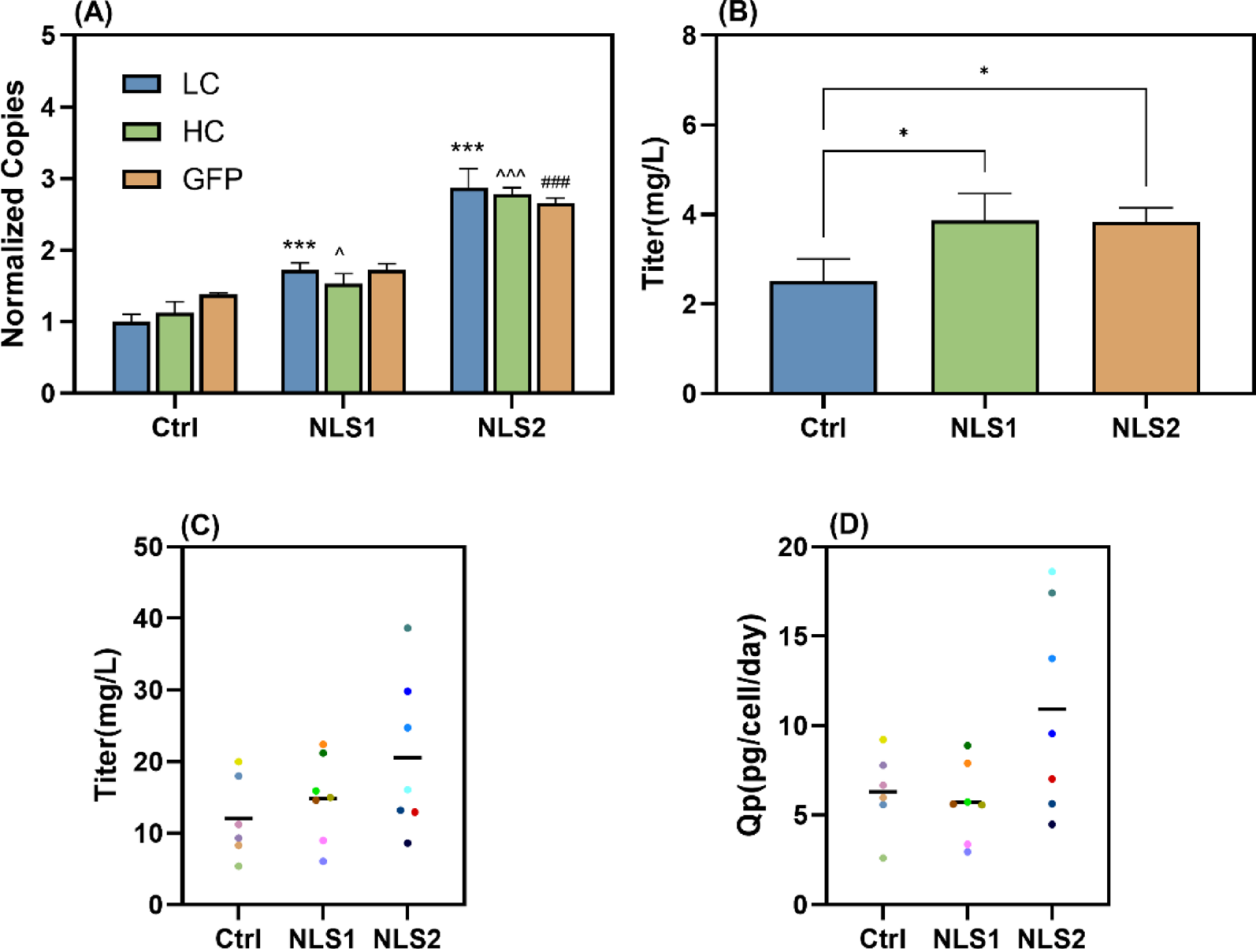
Evaluation of NLS1 and NLS2 effects on gene copies and titer under 10 µg/mL puromycin screening. To analyze mAb production, the recombinant cell pool and clonal cell lines were grown for 4 days in a 24-well plate. **A** Gene copies, **B** Titer (recombinant cell pool), **C** Titer (clone), **D** Qp (clone). The color of each clone’s titer and Qp corresponds to each other, and the black solid line represents the average titer and Qp of the clones in each group. *P*-values were estimated by two-way ANOVA (A-LC-NLS1 and NLS2 vs. Ctrl: *p* < 0.001, *p* < 0.001; HC-NLS1 and NLS2 vs. Ctrl *p* = 0.04, *p* < 0.001; GFP-NLS1 and NLS2 vs. Ctrl: *p* = 0.11, *p* < 0.001) and one-way ANOVA (B-NLS1 and NLS2 vs. Ctrl: *p* = 0.03, *p* = 0.04). ^*^Indicates significant analysis in LC copies and product expression, ^^^indicates significant analysis in HC copies, and ^#^indicates significant analysis in GFP copies between groups

To evaluate the impact on bioproduction, we applied the NLS1 and NLS2 optimized transposon system to mAb production and compared their performance against a no-NLS control. After selection at 10 µg/mL puromycin, we also performed clonal sorting on the recombinant pools and selected the top-GFP clones (6–7 per group) for 4-day batch culture in a 24-well plate. The results demonstrated that NLS1 and NLS2 optimization led to recombinant pools with substantially increased transgene integration (Fig. 6A), resulting in corresponding 95% and 97% enhancements in mAb expression (Fig. 6B). The clones exhibited differential effects: NLS1 slightly elevated average mAb concentration but showed no specific antibody production rate (Qp) improvement, whereas NLS2 enhanced both parameters, with the top producer reaching 38.6 mg/L and 17.41 pg/cell/day (Fig. 6C–D).

### Analysis of the correlations for Qp in cell lines

**Fig. 7:**
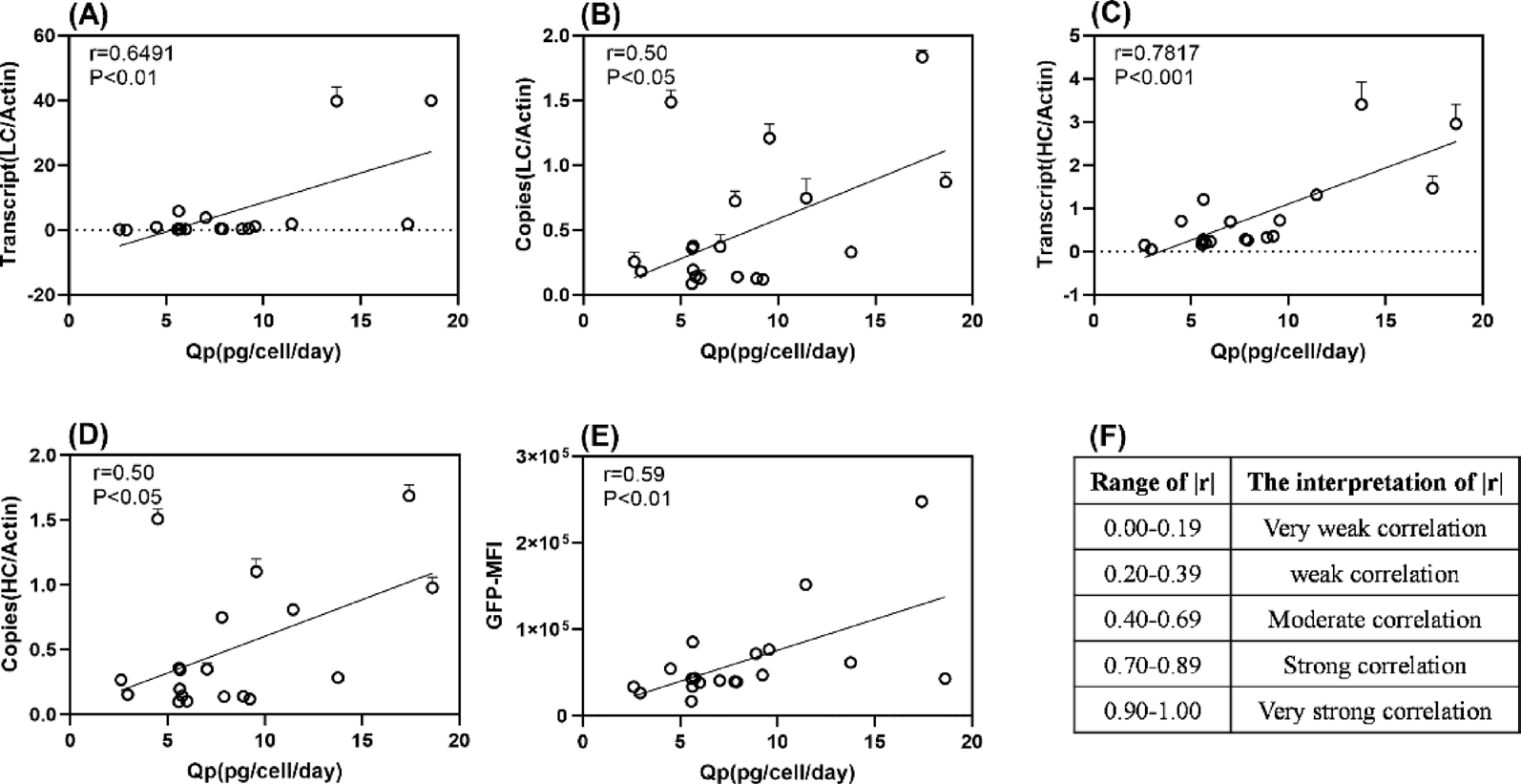
Correlation between gene integration, transcription, GFP-MFI, and Qp. **A** LC-mRNA levels, **B** LC-copies, **C** HC-mRNA levels, **D** HC-copies, **E** GFP-MFI, **F** |r| measures the strength of the linear association between two variables

Through multi-level profiling of integration, transcription, and expression across diverse clones, we established robust correlations and identified key predictive indicators for clonal performance. The results identified a strong correlation between HC mRNA levels and Qp. Further analyses revealed that LC mRNA levels, the LC and HC copies, and GFP-MFI exhibited moderate correlations with Qp (Fig. 7). Consequently, these parameters can serve as effective indicators for evaluating clone expression potential, enabling the selection of high-performing clones.

### Optimized system performance in suspension CHO-K1 cells

**Fig. 8:**
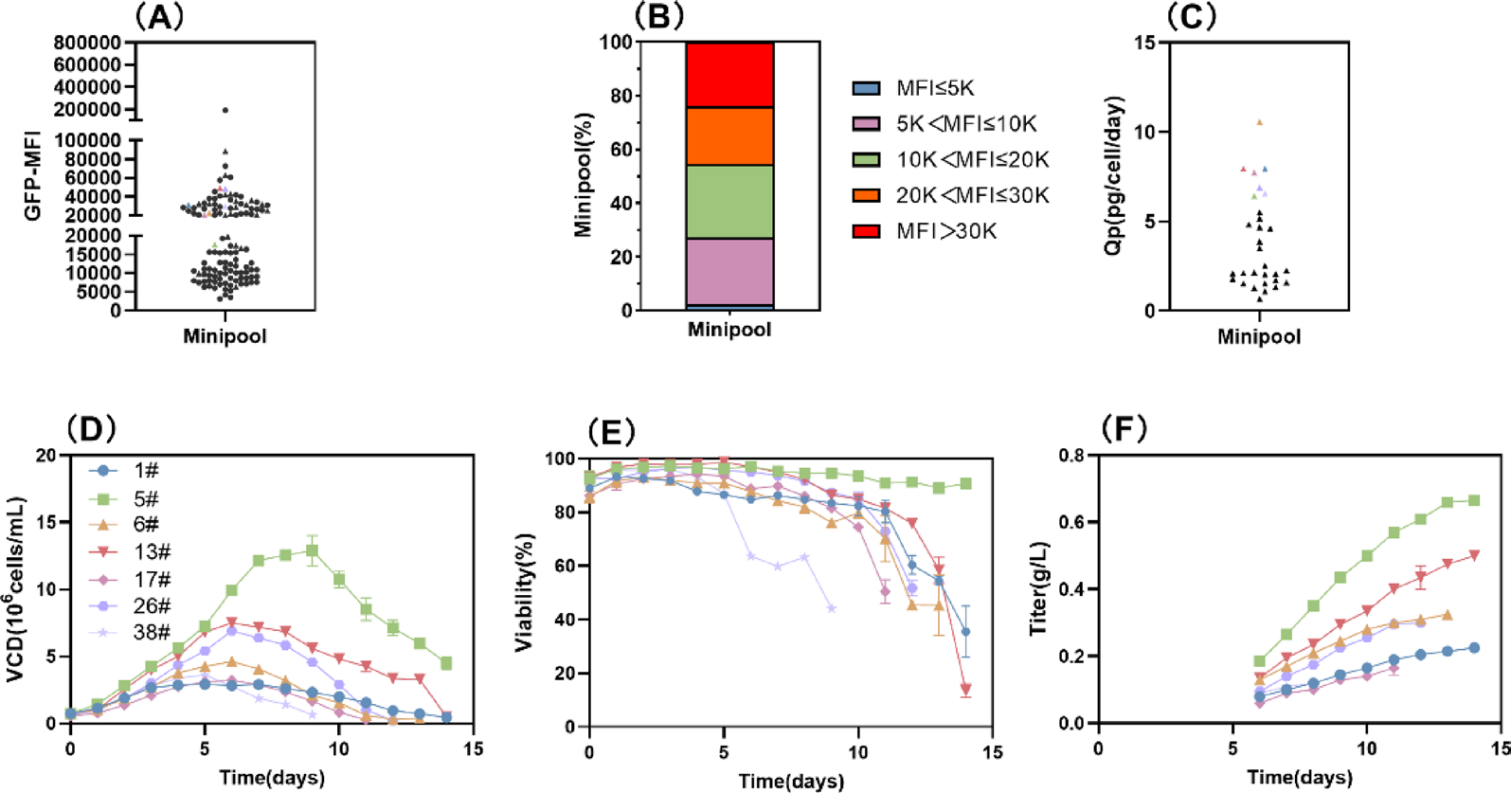
Establishment and fed-batch culture of suspension MP cells. **A** GFP-MFI, **B** The proportion of GFP expression levels of MPs, **C** Qp, **D** Viable Cell Density, **E** Viability, **F** Titer. ▲ represents MPs subjected to fed-batch culture

To evaluate broader applicability, we transitioned the optimal NLS2 systems to suspension cells, seeding them at 2,000 cells/well (MiniPool, MP) in 240 wells of 96-well plates and treating with 10 µg/mL puromycin for selection, with a no-NLS system serving as the control. The results revealed a striking contrast: while only two control MPs showed transient growth before dying, the NLS2 group produced 170 growing MPs. From these, 121 MPs proliferated sustainedly, with 95% showing near-uniform (> 99%) GFP expression (data not shown). The MPs stratified into two distinct populations at the MFI-20 K threshold. Based on standard MFI criteria, high-expression (> 30 K) MPs comprised 23.97%, moderate-high-expression (20–30 K) 21.49%, and low-expression (≤ 5 K) 2.48% (Fig. 8A–B). MPs selected for high MFI and sustained growth underwent a 14-day fed-batch culture, yielding 7 MPs with a Qp exceeding 6 pg/cell/day, including a top performer reaching 10.55 pg/cell/day. However, physiological limitations in growth and maintenance characteristics restricted this MP’s titer to 0.33 g/L despite its high cellular productivity (Fig. 8C–F). Therefore, the optimized system achieved a success rate exceeding 50% for robust MP growth, while the control group failed entirely under identical conditions.

## Discussion

This study demonstrates that incorporating a NLS into the PB transposon system enhances recombinant cell stability and increases protein production. The improved performance was evidenced by a marked rise in the proportion of Class I clones and high-expression clones, indicating the potential of this strategy to streamline cell line screening and substantially improve selection efficiency. The optimized PB system enhanced mAb production at both pool and clonal levels, achieving 97% higher pool expression and generating top clones with Qp of 17.41 pg/cell/day.

Earlier study has indicated that the PB transposon system enables a comprehensive leap in performance: it enhances stable cell line generation efficiency by 15–20 fold and elevates expression levels by 5-fold compared to transient transfection. Furthermore, it ensures markedly improved stability, with a coefficient of variation (CV) of 13–17% versus 21–23% in controls, and has been shown to respond positively to intensified selection pressure (Mattia Matasci et al. 2011), while the inherent biological differences between the cell lines have not been systematically examined. Our research demonstrated that PB transposon and intensified selection improved clonal stability, indicated by increased Class I and decreased Class V/VI clones. However, these strategies showed divergent effects at the population level, where the proportion of positive cells in recombinant pool showed no significant improvement. This is likely attributable to the competitive proliferation advantages held by unstable clones in heterogeneous cultures environments (Matasci et al. 2008).

Additionally, we observed that elevated selection pressure enhanced transgene expression in the PB system without increasing copy numbers-a response distinct from the copy number-dependent behavior of the pcDNA3.1 system. This observation aligns with the established mechanistic model: while random integration (e.g., pcDNA3.1) tends to generate multi-copy clusters at a single locus, the PB transposon distributes copies linearly across distinct genomic sites (Huhn et al. 2023). This suggests that the copy number of the PB transposon system is likely constrained by the saturation of available genomic integration sites.

Recent studies have highlighted the potential of subcellular targeting strategies to optimize transposon-mediated gene delivery. The combination of PBase with an HIV Rev-derived nucleolar localization signal (NoLS) significantly enhanced transposition efficiency and increased transgene copy numbers through a nucleolar targeting strategy (Solenne Bire et al. 2021). Building on the foundational principle of subcellular targeting to optimize genome-editing tools, our study systematically evaluated NLS variants and demonstrated for the first time that strategic fusion of an NLS to the transposase not only influences nuclear import but, more importantly, profoundly enhances the long-term stability of recombinant cell lines-a critical and often limiting factor in bioproduction. Through comprehensive comparative screening, the nucleoplasmin-derived NLS emerged as the most potent variant. It consistently improving stability approximately two-fold across selection pressures, whereas a modified c-myc NLS (Mc-myc) showed only marginal benefit, highlighting the functional specificity of NLS sequences. The advantage of the nucleoplasmin NLS extends beyond stability. Unlike the histone H2B-Tol2 system which was reported to increase average expression without improving the yield of top producers (Park et al. 2022), the nucleoplasmin NLS delivered a comprehensive performance enhancement. It boosted the average antibody titer by 40% while simultaneously doubling the expression level of the highest-producing clones. Remarkably, this NLS variant enabled sustained high-level expression with superior per-copy productivity under two distinct selection pressures. The functional profile demonstrates that the nucleoplasmin NLS consistently generates integration events capable of robust expression across both production environments. Therefore, the stability and per-copy expression advantages conferred by nucleoplasmin NLS are not merely phenotypic traits but translate into tangible process benefits. This is clearly evidenced in suspension cells, where the nucleoplasmin NLS achieved a 50% higher efficiency in recombinant cell pool generation. This result demonstrates that the utility of our strategy extends beyond enhancing product titer and clone stability; it directly addresses the persistent practical bottleneck of low transfection and stable integration efficiency in hard-to-manipulate cells, thereby streamlining the entire cell line development pipeline.

Beyond identifying a superior standalone NLS, our study also sought to define the functional repertoire of different NLS motifs to inform more sophisticated engineering. Our analysis further identifies the classic SV40 NLS as a versatile, dual-function module. It confers robust stability under high selection pressure while maintaining favorable per-copy productivity and appears to facilitate initial integration events under low-pressure conditions. Building on our study which delineates the functional profile of SV40 NLS within the PB system and its established historical role in enhancing transposition efficiency (Charng et al. 2004), we propose a synergistic combination of SV40 NLS with nucleoplasmin NLS as a rational design strategy for next-generation cell line engineering. This integrated strategy is designed to harness the complementary strengths of both NLS signals-leveraging SV40’s efficiency in promoting integration and nucleoplasmin’s precision in directing genomic targeting-to establish a robust and versatile platform for engineering high-yield, stable recombinant cell lines for biomanufacturing applications.

## Conclusion

We establish that the coupling of NLS to PB transposase is a novel and effective strategy for enhancing the stability of recombinant cell pools. Specifically, *Xenopus laevis* nucleoplasmin provided comprehensive enhancement of cell line stability regardless of selection stringency. This effect was further enhanced under high-selection pressure, which drove the robust expansion of Class I and other high-expression clone populations. Our results demonstrate that transposase-nucleoplasmin efficiently streamlines cell line development. Looking forward, protein engineering of nucleoplasmin to enhance its nuclear targeting activity or compatibility with other NLSs could yield even more powerful hyper-active transposase variants.

## Supplementary Information

Below is the link to the electronic supplementary material.

Supplementary Material 1

## Data Availability

Data will be made available on reasonable request.

## Abbreviations

CHO: Chinese hamster ovary
NLS: Nuclear localization signal
RTI: Random transgene integration
STI: Semi-targeted transgene integration
SSI: Site-specific transgene integration
ITRs: Inverted terminal repeats
PBase: PiggyBac transposase
mAb: Monoclonal antibody
MFI: Mean fluorescence intensity
copGFP: Codon-optimized green fluorescent protein
mCherry: Monomeric cherry fluorescent protein
IRES2: Internal ribosome entry site 2
pac: Puromycin N-acetyl-transferase gene
CMV: Mouse cytomegalovirus major immediate early promoter/enhancer
PBS: Phosphate-buffered saline
ELISA: Enzyme-linked immunosorbent assay
µ: Specific growth rate
Qp: Specific antibody production rate
