# Supplementary material for: Engineered NLS-PBase system boosts stability and productivity in recombinant cell lines
Source: Bioresour Bioprocess. 2026 Apr 28;13(1):59. doi: 10.1186/s40643-026-01056-x (PMC13125444; doi:10.1186/s40643-026-01056-x)
Supplement: Supplementary file 1 — Supplementary Material 1 [file 40643_2026_1056_MOESM1_ESM.docx]

Supplementary Materials

**Table. S1 Assignment of Specific Growth Rates (μ) to Cell Line Categories I-VI**

| **Categories** | **GFP (%)** | **μ (Day^-1^)** | **Mean (GFP%)** |
| --- | --- | --- | --- |
| Ⅰ | 99-100 | 0.53 | 99.5 |
| Ⅱ | 95-99 | 0.54 | 97 |
| Ⅲ | 75-95 | 0.57 | 85 |
| Ⅳ | 50-75 | 0.57 | 62.5 |
| Ⅴ | 10-50 | 0.6 | 30 |
| Ⅵ | 0-10 | 0.6 | 5 |


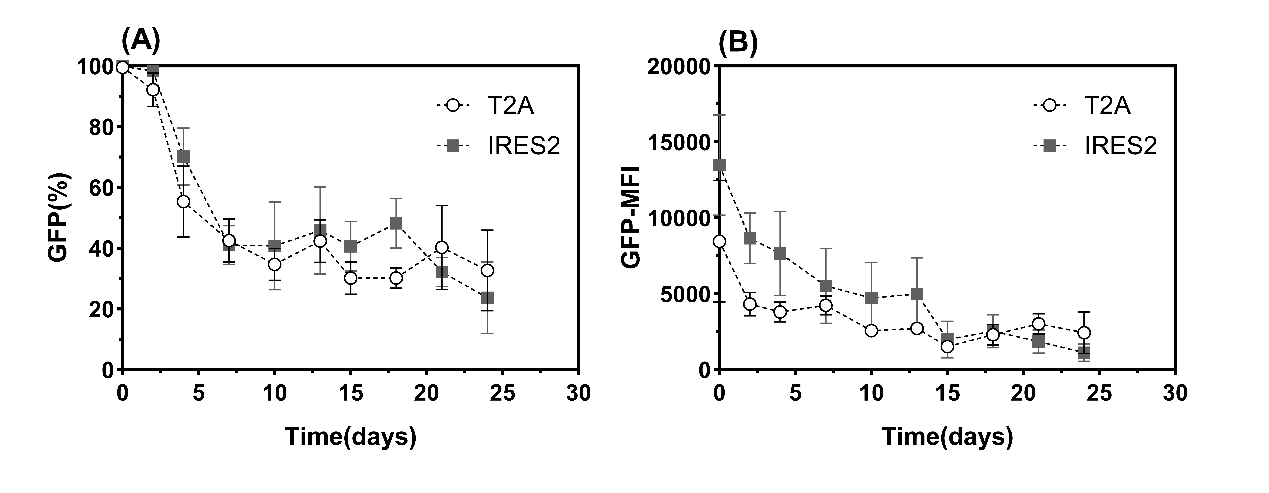


Fig. S1. The positive cell percentage and expression analysis of recombinant cell pools generated by T2A and IRES2 linkers during culture without selection pressure. (A) The positive cell ratio. (B) GFP-MFI.


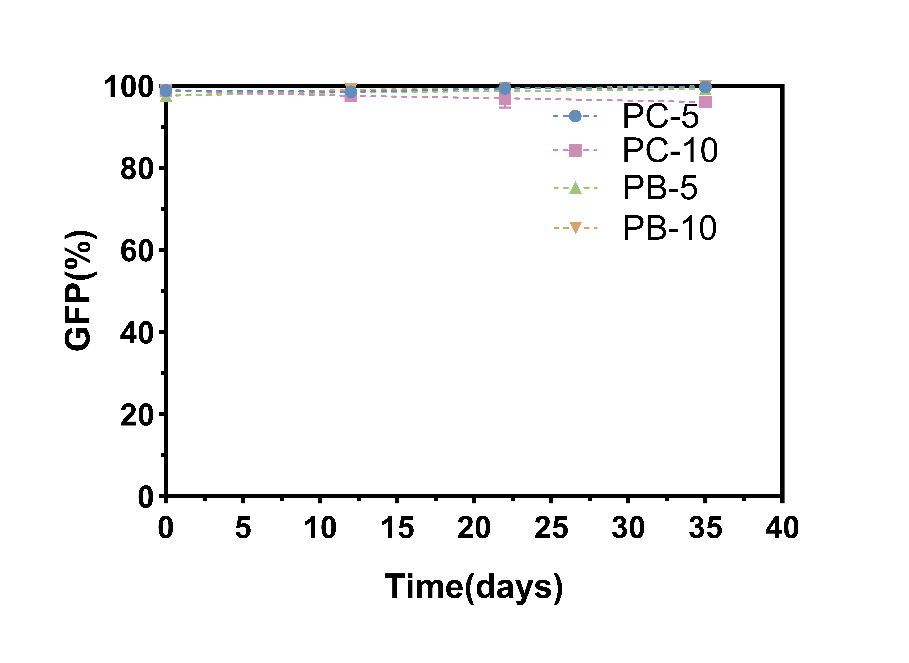


Fig. S2. Positive cells in recombinant pools: screening intensity and vector effects under selection. The stable transfected recombinant cell pools established with pcDNA3.1 at 5 μg/mL and 10 μg/mL puromycin are designated as PC-5 and PC-10; the stable transfected recombinant cell pools established with PiggyBac at 5 μg/mL and 10 μg/mL puromycin are designated as PB-5 and PB-10.


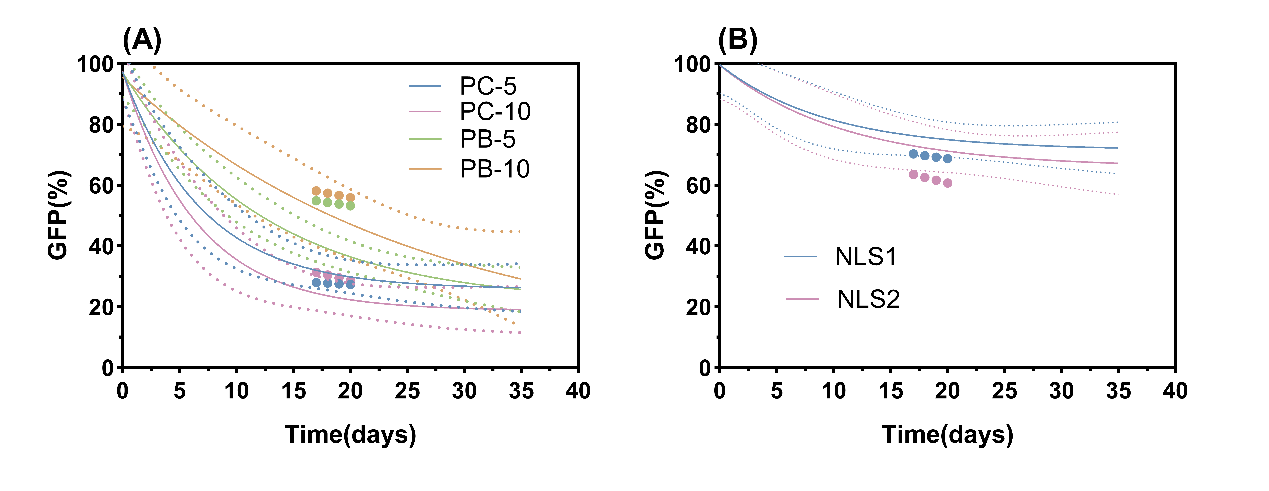


Fig. S3 Reverse inference of cell-pool heterogeneity from clonal cell lines. A The fitting of predicted versus actual values for recombinant cell pools under 5 μg/mL and 10 μg/mL puromycin selection for pcDNA3.1 and PiggyBac, B The fitting of predicted versus actual values for recombinant cell pools under 10 μg/mL puromycin selection for the NLS1 and NLS2 groups. The solid line represents the change in the proportion of positive cells during the culture process as the actual value, the dashed line represents the 95% confidence interval range, and the solid points represent the predicted proportion of positive cells in the cell pool from D17 to D20 by the cell line.
